# Supplementary figures and images for: Research Trends and Hotspots Analysis Related to Monocarboxylate Transporter 1: A Study Based on Bibliometric Analysis
Source: Int J Environ Res Public Health. 2019 Mar 27;16(7):1091. doi: 10.3390/ijerph16071091 (PMC6479916; doi:10.3390/ijerph16071091)

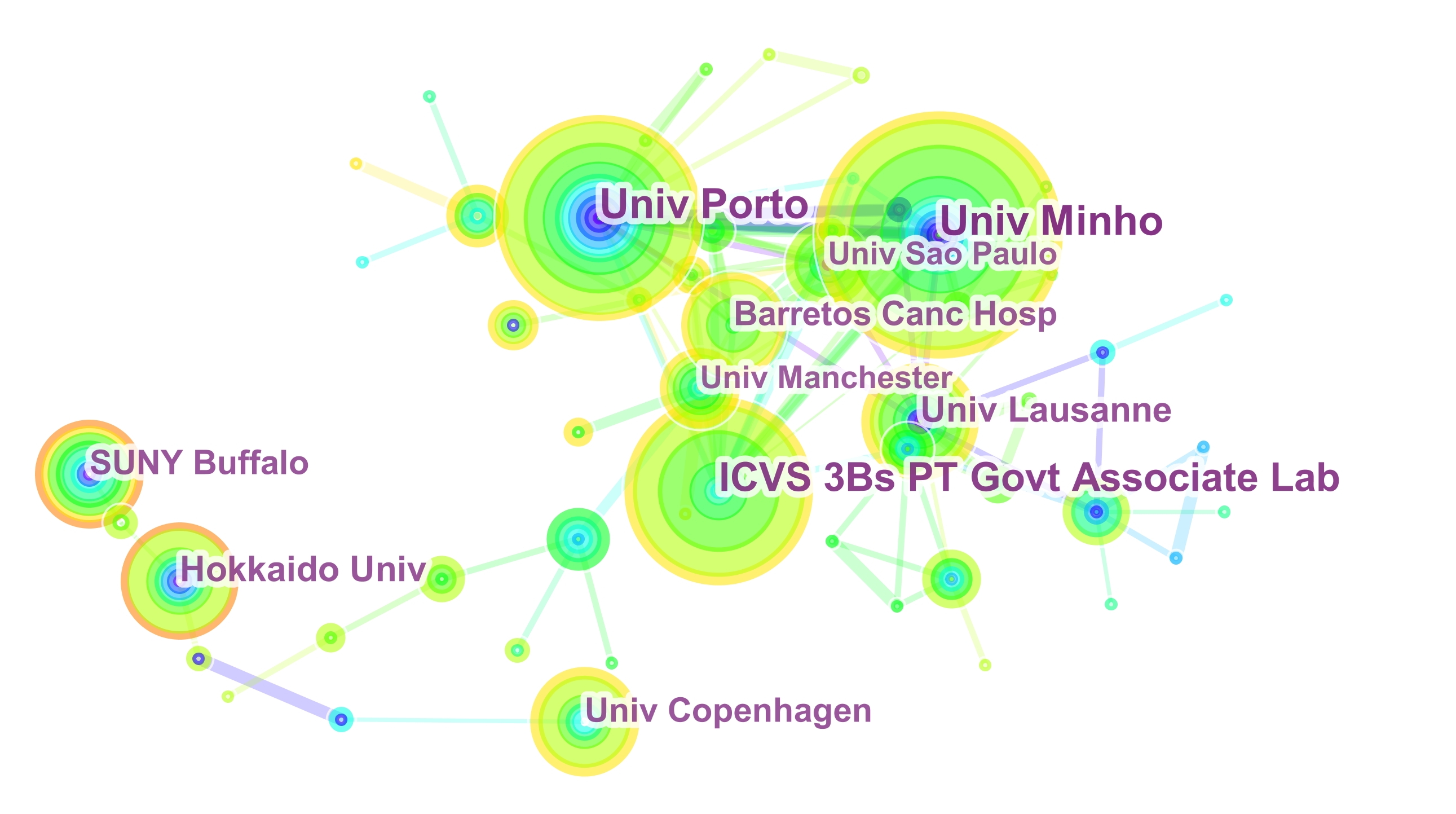

Supplement: Supplementary file 1 [file ijerph-16-01091-s001.zip › Supplementary Files/Figure S1.jpg]

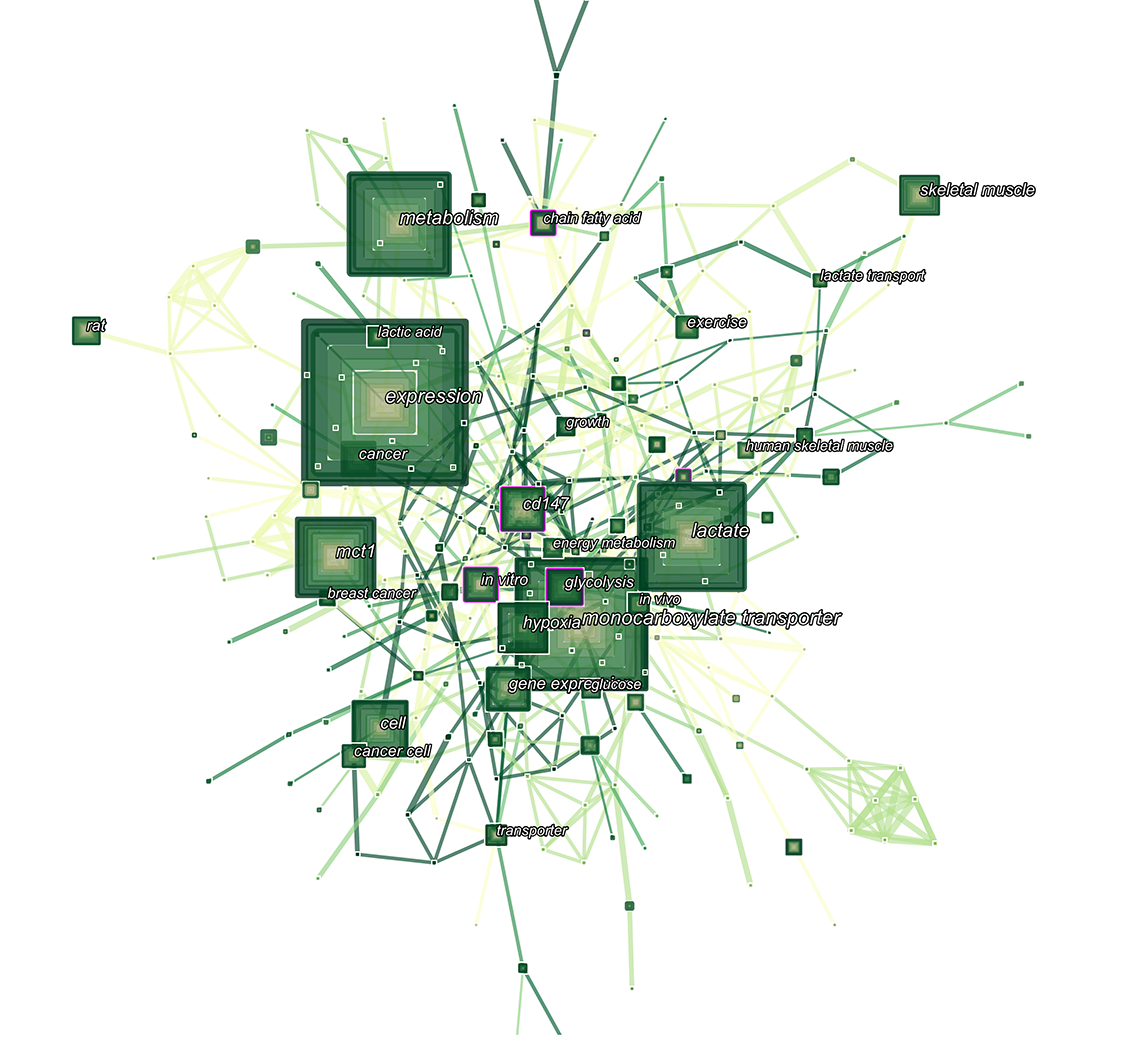

Supplement: Supplementary file 1 [file ijerph-16-01091-s001.zip › Supplementary Files/Figure S2.png]

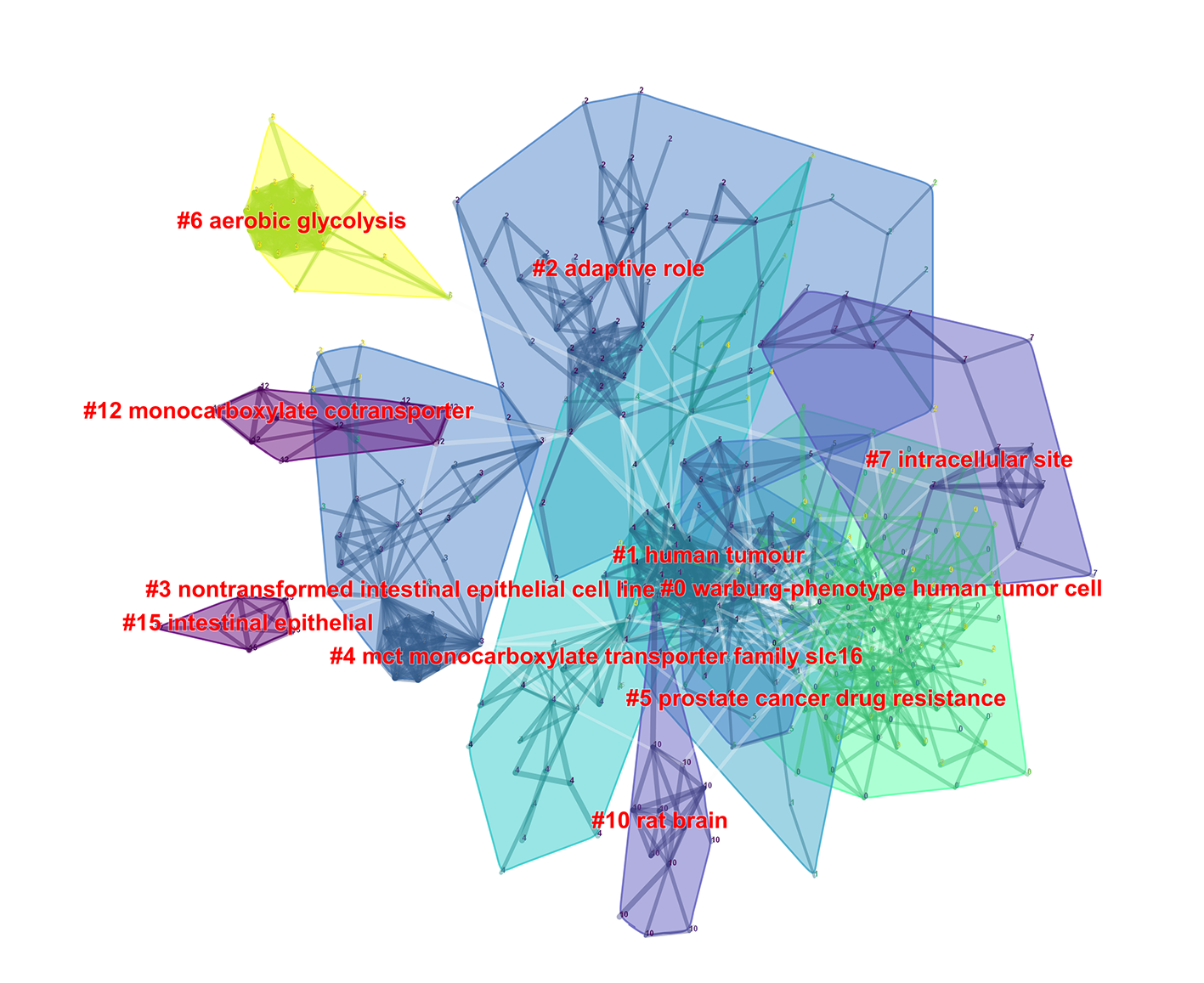

Supplement: Supplementary file 1 [file ijerph-16-01091-s001.zip › Supplementary Files/Figure S3.png]
